# Investigation of the association between the triglyceride-glucose index and the incidence of frailty among middle-aged and older adults: evidence from the China health and retirement longitudinal study

**DOI:** 10.3389/fpubh.2025.1548222

**Published:** 2025-04-30

**Authors:** Qingwen Long, Yongli Li, Zijuan Shi, Yujun Lee, Lifang Mao

**Affiliations:** ^1^Department of Nursing, Affiliated Hospital of North Sichuan Medical College, Nanchong, China; ^2^Department of Nursing, North Sichuan Medical College, Nanchong, China

**Keywords:** frailty, frailty index, middle-aged and older adults, triglycerides-glucose index, CHARLS

## Abstract

**Aim:**

Limited researches have investigated the association between the Triglyceride-glucose index (TyG) and frailty vulnerability in middle-aged and older Chinese populations. This study aims to strengthen the scientific foundation for frailty prevention and management by analyzing the correlation between TyG and frailty, ultimately providing more targeted intervention strategies.

**Methods:**

This study included middle-aged and older individuals who participated in the China Health and Retirement Longitudinal Study (CHARLS) from 2015. A multiple logistic regression model was constructed to assess the correlation between the TyG index and frailty. Additionally, restricted cubic spline (RCS) analysis was employed to evaluate the dose–response correlation between the two variables.

**Results:**

Among the 3,978 participants included in the study, 667 individuals (16.8%) were identified with frailty. After adjusting for multiple factors in a logistic regression model, it was determined that individuals in the highest quartile group of the TyG index had a 1.43 times higher risk of frailty compared to those in the lowest quartile group (OR = 1.43, 95% CI: 1.10 ~ 1.85, *p* = 0.007). The RCS analysis further revealed a positive dose–response relationship, indicating that a higher TyG index was associated with an increased risk of frailty in middle-aged and older adults.

**Conclusion:**

Research has shown a significant positive linear relationship between an increased TyG index and a higher prevalence of frailty in middle-aged and older individuals. Elevated TyG index levels could signal an increased vulnerability to frailty among individuals.

## 1 Introduction

Frailty, a multidimensional physiological condition, is marked by diminishing reserves and functions of various organ systems, weakened stress responses, increased vulnerability to diseases, and increased mortality risks (1, 2). It is commonly observed in middle-aged and older populations as they advance in age (3). As the population ages at a faster rate, frailty affects approximately 10% of community-dwelling older adults aged 60 and above, 15% for individuals aged 75–84, and 25% for those aged 85 and older in China (4, 5). In the world, the prevalence of frailty among individuals aged 50 and older is 24% across 62 countries or regions worldwide (6). Frailty is not a distinct disease but a constellation of symptoms and indicators potentially leading to disabilities, falls, depression, and reduced quality of life (7, 8). Moreover, it represents a significant contributor to mortality among middle-aged and older adults, placing substantial burdens on healthcare systems worldwide (9). However, frailty represents an early and reversible condition (3). Early identification of risk factors, regular frailty screening, and appropriate interventions are crucial for preventing and managing frailty. These measures are poised to significantly impact clinical practice and public health initiatives (3, 8, 9).

The syndrome of frailty is complex and influenced by multiple factors, sharing common pathogenic mechanisms with sarcopenia. Patients with muscle-wasting disorders often experience conditions with muscle strength and mass, which directly impacts physical function and increases vulnerability to frailty (10, 11). Consequently, mitigating and managing frailty primarily involves preventing the decline in muscle mass and function. Previous research has shown a strong correlation between insulin resistance (IR) and the decrease in muscle mass, along with the deterioration of muscle function (12, 13). IR is characterized by a decreased sensitivity of the body to insulin, leading to impaired glucose uptake and utilization (14, 15). A reduction in IR may cause muscle wasting and reduced strength, thereby worsening frailty. Studies have identified potential pathways linking IR and frailty, including muscle metabolism, inflammation, and oxidative stress (16, 17). The Triglyceride-glucose index (TyG), an emerging potential marker for IR, is significantly associated with the reduction of muscle mass and strength among older individuals (18–20). Research also demonstrates a direct relationship between the TyG index and sarcopenia, suggesting that elevated TyG index levels correspond to an increased risk of developing sarcopenia (21).

Compared to traditional approaches for measuring insulin, the TyG index offers advantages including operational convenience, quick reporting, and lowered economic impact (22). Researches have revealed a significant association between the TyG index and the onset of various chronic conditions, such as coronary artery disease, ischemic stroke, heart failure, chronic kidney disease, hypertension, and diabetes (21, 23–27). These studies show the value of the TyG index in predicting the risk of cardiovascular diseases, hypertension, diabetes, and sarcopenia. Furthermore, it is suggested that the TyG index may play an indirect role in the development of frailty.

With advancing age, bodily systems gradually deteriorate, leading to an increased risk of frailty. Aging also increases IR, with the TyG index serving as a marker of IR, thereby influencing the development of frailty (28). However, the association between the TyG index and frailty has been explicitly addressed in only two studies. These studies have shown that persistently elevated TyG index levels may have a potential impact on identifying older individuals at increased risk of frailty (29, 30). However, these studies are limited by their participant selection, predominantly concentrating on specific regions and ethnicities, thereby limiting their generalizability to the wider middle-aged and older population in China. Consequently, this study made use of the CHARLS database, which pertains to the middle-aged and older population in China, to examine the association between the TyG index and frailty. The purpose is to provide evidence supporting the prevention and management of frailty development.

## 2 Methods

**Figure 1:**
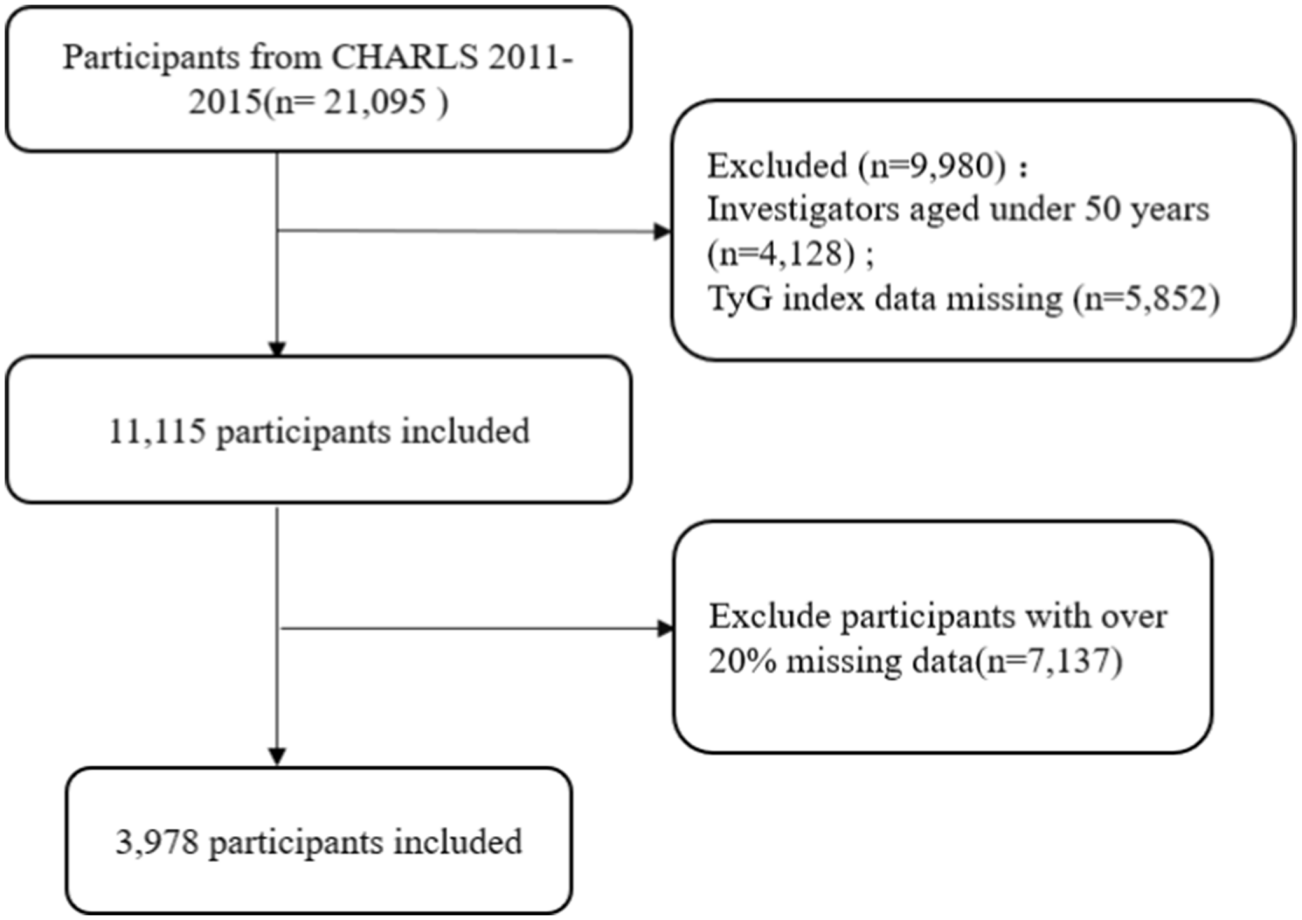
A flow diagram depicting the exclusion criteria for research participants.

Data from the China Health and Retirement Longitudinal Study (CHARLS) database were obtained for this study.^1^ CHARLS, a longitudinal study in mainland China targeting individuals aged 45 and older with a multi-stage sampling across 150 counties and 450 villages, with follow-ups conducted every 2–3 years (31). The participants in the CHARLS survey all provided written informed consent, and the survey obtained approval from the Peking University Biomedical Ethics Committee (No. IRB00001052-11015). We accessed data from the 2015 CHARLS after receiving approval from Peking University’s National School of Development. Participants younger than 50 or those with missing TyG index or frailty data were excluded from the data, resulting in a final sample of 3,978 participants, as shown in Figure 1.

## 3 Measures

### 3.1 Frailty

The frailty phenotype (FP) and frailty index (FI) are two main approaches used to evaluate frailty (32). Since the FP is considered more appropriate for older inpatients, this study adopted the FI developed by Searle et al. (33). An FI index was constructed using 36 health-related items, covering cognitive function, activities of daily living (ADL), instrumental activities of daily living (IADL), common chronic diseases (diabetes, stroke, heart disease, etc.), mental health, and sensory impairments. Detailed information on these items can be found in Supplementary Table 1. Each indicator was scored as 1 if the health standard was met and 0 otherwise. The FI was calculated by dividing the total impairment score by 36, yielding a value between 0 and 1. Based on previous researches (34), frailty is defined as having an FI ≥ 0.25, with higher FI scores indicating greater frailty severity.

### 3.2 TyG

Participants underwent blood tests of fasting triglyceride and blood glucose, which were used to calculate the TyG index using the formula: ln[fasting triglycerides (mg/dl) × fasting blood glucose (mg/dl)/2] (22). The fasting period was at least 8 h.

### 3.3 Covariates

This study considered covariates such as sociodemographic characteristics (age, gender, residential area, educational attainment, marital status), health behaviors (smoking status denotes the current smoking status, encompassing both daily and occasional smokers. Alcohol intake refers to the current drinking status, characterized by a consistent pattern of alcohol consumption.), physical indices (waist circumference, body mass index), and biochemical markers (hemoglobin, high-density lipoprotein, low-density lipoprotein, total cholesterol) to account for potential influences on the relationship between the TyG index and frailty.

### 3.4 Statistical analysis

Categorical variables are shown as frequencies and percentages (n %), analyzed with the χ^2^-test. Continuous variables are described by median and quartiles (M, P25, P75) and were compared using the Mann–Whitney U test. The relationship between the TyG index and the occurrence of frailty is analyzed through binary logistic regression when the index is viewed as a continuous variable. Through the utilization of quartiles of the TyG index, three logistic regression models were established to assess the risk of frailty. The outcomes were illustrated using odds ratios (OR) and 95% confidence intervals (95% CI). Model 1 serves as the initial coarse adjustment mode. Model 2 further refines the analysis by incorporating adjustments for age, gender, place of residence, and educational level. Moreover, Model 3 expands the scope of adjustments to include smoking, alcohol consumption, waist circumference, and BMI. All variables considered in the model adjustment were subsequently analyzed using the correlation matrix and multicollinearity tests. After adjusting for all covariates, we used the Restricted Cubic Spline (RCS) analysis to examine the dose–response relationship between the TyG index and frailty. Then, subgroups based on age, gender, and BMI index were assessed to examine potential interactions with the TyG index. Statistical analyses were performed utilizing R 4.4.0 and SPSS 26.0, with a significance level established at *α* = 0.05.

## 4 Results

### 4.1 Baseline features of individuals

With a total of 3,978 participants, the study consisted of 1,965 males (49.4%) and 2,013 females (50.6%), with a median age of 60 years, 16.8% (667 individuals) were found to have experienced frailty. Upon stratifying research participants according to frailty levels for comparative baseline characteristic analysis, it was evident that the frail cohort had a higher proportion of females relative to males, as well as being older, having elevated BMI, waist circumference, and TyG index. Furthermore, frail individuals residing in rural areas exhibited a heightened vulnerability to frailty compared to urban dwellers, with lower levels of education, high-density lipoprotein cholesterol, and hemoglobin. These differences were statistically significant, as presented in Table 1.

**Table 1:** The baseline features of the research subjects were compared through analysis.

Variables	Total (*n* = 3,978)	Frailty (*n* = 667)	Non-frailty (*n* = 3,311)	χ^2^/Z	*P*
Age	60(54 66)	62(57 67)	60(53 65)	−7.773	< 0.001
Gender (%)				36.815	< 0.001
Male	1,965(49.4)	258(38.7)	1,707(51.6)		
Female	2,013(50.6)	409(61.3)	1,604(48.4)		
Education (%)				38.368	< 0.001
Illiterate	434(10.9)	112(16.8)	322(9.7)		
Below junior high school	2,921(73.4)	484(72.6)	2,437(73.6)		
Above junior high school	623(15.7)	71(10.6)	552(16.7)		
Marital status (%)				1.867	0.172
Married	3,599(90.5)	594(89.1)	3,005(90.8)		
Unmarried/divorced/widowed	379(9.5)	73(10.9)	306(9.2)		
Place of residence (%)				5.568	0.018
Urban	1,239(31.1)	182(27.3)	1,057(31.9)		
Rural	2,739(68.9)	485(72.7)	2,254(68.1)		
Current smoking status (%)				10.195	0.001
Non-smoking	2,114(53.1)	392(58.8)	1,722(52)		
Smoking	1,864(46.9)	275(41.2)	1,589(48)		
Current drinking status (%)				41.759	< 0.001
Non-drinking	2,527(63.5)	497(74.5)	2,030(61.3)		
Drinking	1,451(36.5)	170(25.5)	1,281(38.7)		
BMI (%)				9.227	0.002
≤24 kg/m^2^	1,919(48.2)	286(42.9)	1,633(49.3)		
>24 kg/m^2^	2,059(51.8)	381(57.1)	1,678(50.7)		
HDL	49.4(42.5 57.1)	48.7(41.7 56)	49.4(42.5 57.1)	−2.436	0.015
LDL	101.5(83.7120.8)	101.5(81.5121.2)	101.5(83.8120.5)	−0.370	0.712
CHO	182.6(161.4206.9)	183(161.4207.7)	182.6(161207)	−0.350	0.727
HGB	13.8(12.7 14.9)	13.4(12.4 14.6)	13.9(12.8 15)	−5.344	< 0.001
WC	87.4(80.4 94.6)	89.5(82 97.4)	87(80 94)	−5.640	< 0.001
TyG	8.68(8.29 9.15)	8.8(8.39 9.26)	8.65(8.28 9.12)	−4.434	< 0.001
FI	0.16(0.11 0.22)	0.30(0.27 0.35)	0.15(0.10 0.19)	−40.806	< 0.001

BMI, body mass index (≤24 kg/m^2^, >24 kg/m^2^); HDL, high-density lipoprotein; LDL, low-density lipoprotein; CHO, total cholesterol; HGB, hemoglobin; WC, waist circumference; TyG, triglyceride-glucose index; FI, frailty index.

### 4.2 Association between TyG index and frailty

**Figure 2:**
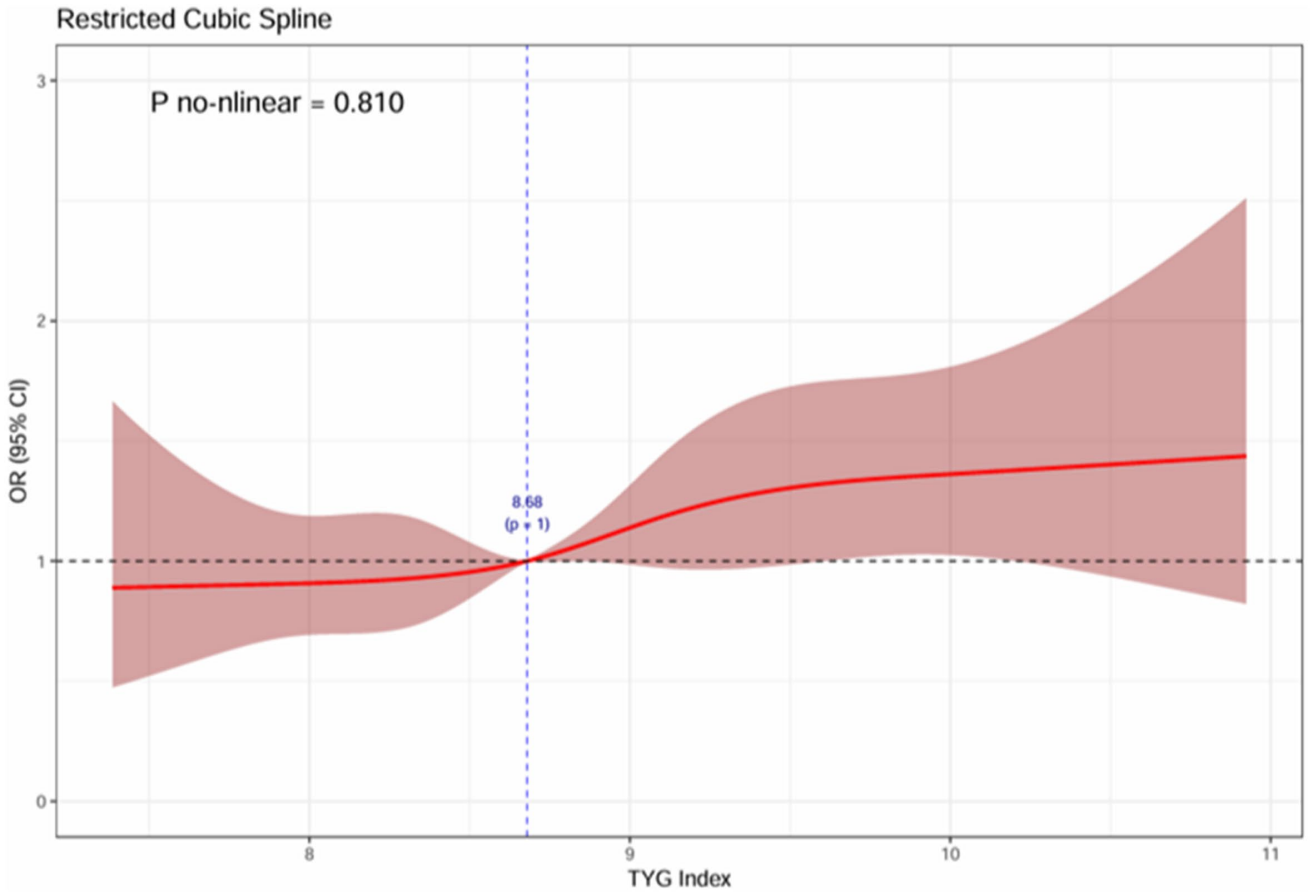
The association between TyG and the dose–response of frailty.

The analysis of three logistic regression models detailed in Table 2 revealed a significant positive relationship between higher TyG index values and an increased incidence of frailty. In the unadjusted crude model 1, the risk of frailty occurrence was 1.57 times higher in the highest quartile (Q4) of the TyG index group compared to the lowest quartile (Q1) group (OR = 1.57, 95%CI: 1.24 ~ 1.99). Within model 3, after adjusting for covariates like gender, age, residence, education level, smoking habits, alcohol consumption, hemoglobin levels, waist circumference, and BMI, it was observed that individuals positioned in the highest quartile (Q4) of the TyG index were more likely to experience frailty than those placed in the lowest quartile (Q1) (TyG Q4 vs. Q1, OR = 1.43, 95%CI: 1.10 ~ 1.85, *p* = 0.007). The correlation matrix and multicollinearity tests showed that the variance inflation factors (VIFs) for all variables in the fully adjusted model were less than 2. Detailed findings can be found in Supplementary Figures 1–4. After adjusting for all covariates, employing RCS analysis showed a significant dose–response relationship between the TyG index and the occurrence of frailty. The risk of frailty demonstrates a slow escalation with TyG index values up to 8.68, at which point it commences a gradual ascent. A higher TyG index correlated with an elevated risk of frailty, suggesting a linear positive association between the TyG index and the risk of frailty (see Figure 2).

**Table 2:** The results of logistic analysis between TyG and frailty.

Variables	Model1		Model 2		Model 3	
	OR(95% CI)	*P-*value	OR(95% CI)	*P*-value	OR(95% CI)	*P*-value
TyG (continuous)	1.32 (1.17 ~ 1.50)	< 0.001	1.36 (1.20 ~ 1.55)	< 0.001	1.26 (1.10 ~ 1.45)	0.001
Group						
Q1	Ref		Ref		Ref	
Q2	1.04 (0.81 ~ 1.34,)	0.763	1.02 (0.79 ~ 1.31)	0.897	0.98 (0.76 ~ 1.27)	0.882
Q3	1.35 (1.06 ~ 1.72)	0.014	1.36 (1.06 ~ 1.74)	0.016	1.22 (0.95 ~ 1.58)	0.121
Q4	1.57 (1.24 ~ 1.99)	< 0.001	1.65 (1.29 ~ 2.10)	< 0.001	1.43 (1.10 ~ 1.85)	0.007

The TyG index, was categorized into four quartiles: Q1 (<8.29 mg/dL), Q2 (8.29–8.68 mg/dL), Q3 (8.68–9.14 mg/dL), and Q4 (≥9.14 mg/dL). Ref: reference. Model 1 was unadjusted, while Model 2 included adjustments for gender, age, residence, and education level. Model 3 expanded these adjustments to incorporate smoking, alcohol consumption, hemoglobin levels, waist circumference, and BMI.

### 4.3 Subgroup analysis and interaction tests

After controlling for confounding variables, a subgroup analysis including gender, age, and BMI was conducted to explore potential interactions between subgroup variables and the TyG index regarding the risk of frailty onset (Table 3). No significant interaction was observed among the subgroups based on gender, age, and BMI, suggesting consistent outcomes.

**Table 3:** Subgroup analysis of frailty with TyG index.

Subgroup	TyG index				P for interaction
	Q1	Q2	Q3	Q4	
Age					0.436
<65 years	Ref	0.84(0.6 ~ 1.17)	1.12(0.81 ~ 1.54)	1.21(0.87 ~ 1.67)	
≥65 years	Ref	1.18(0.78 ~ 1.79)	1.39(0.92 ~ 2.12)	1.9(1.23 ~ 2.93)	
Gender					0.350
Female	Ref	1.2(0.82 ~ 1.76)	1.16(0.78 ~ 1.72)	1.45(0.97 ~ 2.18)	
Male	Ref	0.85(0.59 ~ 1.21)	1.27(0.91 ~ 1.79)	1.44(1.03 ~ 2.03)	
BMI					
≤24 kg/m^2^	Ref	1.1(0.78 ~ 1.54)	1.26(0.87 ~ 1.82)	1.78(1.21 ~ 2.6)	0.576
>24 kg/m^2^	Ref	0.83(0.55 ~ 1.25)	1.13(0.78 ~ 1.64)	1.17(0.81 ~ 1.69)	

The TyG index, was categorized into four quartiles: Q1 (<8.29 mg/dL), Q2 (8.29–8.68 mg/dL), Q3 (8.68–9.14 mg/dL), and Q4 (≥9.14 mg/dL). Ref: reference. BMI, body mass index (≤24 kg/m^2^, >24 kg/m^2^).

## 5 Discussion

In this large-scale, cross-sectional study, we investigated the association between the TyG index and frailty in middle-aged and older Chinese individuals for the first time. The study revealed that 16.8% of participants experienced frailty. After adjusting for confounding factors, individuals in the highest quartile (Q4) of the TyG index showed a 1.43 times higher risk of frailty compared to those in the lowest quartile (Q1), indicating a positive association between elevated TyG index levels and increased frailty occurrence. This association was further supported by the RSC analysis, which demonstrated a linear positive correlation between the TyG index and the risk of frailty development. In the subgroup analysis, there was no interaction observed between gender, age, and BMI with the TyG index, indicating consistent effects across diverse subgroups and thereby strengthening the generalizability and reliability of the study findings.

The current research results suggest a positive association between an increase in the TyG index and the occurrence of frailty, aligning closely with previous study outcomes. Among urban older residents aged 60 and above in a specific region of China, findings from a prospective cohort study suggest that an elevated TyG index and a consistently high trajectory of the TyG index are associated with an increased vulnerability to frailty (29). A recent cross-sectional study of individuals aged 50 and above in the United States revealed a positive association between the elevated TyG index and its related indices with a higher prevalence of frailty (30). There is a lack of research on the correlation between the TyG index and frailty. Previous studies have not determined the link between the TyG index and frailty in the older Chinese population due to geographical and racial diversity constraints. This study used the CHARLS database covering 28 provinces in China. With a sample of 23,000 participants from 12,400 households representing various urban and rural populations, the study aimed to explore the relationship between the TyG index and frailty risk among middle-aged and older individuals in China.

Frailty in the older is characterized by systemic dysregulation affecting multiple systems, leading to reduced physiological reserves, decreased stress resilience, and compromised recovery capacity, highlighting its importance as a significant geriatric syndrome (35, 36). The decline in muscle mass or strength among older adults significantly contributes to frailty. Prior research has suggested that a high TyG index poses a risk for reduced muscle mass or strength, especially in young and middle-aged individuals with hypertension (21, 37). There is a nonlinear correlation between the TyG index and sarcopenia in populations without metabolic syndrome or diabetes and with inadequate physical activity. Li et al. assessed the relationship between TyG index and low skeletal muscle mass and found a significant independent interaction regardless of age and gender (38).

The development and progression of frailty in the older can be affected by numerous factors, with the simultaneous presence of multiple diseases being a key risk factor that speeds up the onset of frailty (39). The TyG index, serving as a reliable surrogate biomarker for IR, is a critical metabolic parameter with demonstrated high specificity and sensitivity in the assessment and prediction of conditions such as hypertension, diabetes, and cardiovascular diseases (21, 22, 26). An increased risk of hypertension is linked to elevated TyG index and its long-term trajectory (40, 41), with the TyG index demonstrating superior predictive value for type 2 diabetes compared to fasting blood glucose and glycated hemoglobin (21, 25, 27). Furthermore, the TyG index is connected to the occurrence of microvascular complications in diabetes (42). The TyG index plays a critical role in the development, advancement, and prediction of cardiovascular diseases, as elevated TyG levels are associated with negative results in individuals with coronary artery disease and increased mortality rates in heart failure patients (26, 40, 43). Age is a non-modifiable factor that impacts the progression of frailty, as bodily systems deteriorate over time, leading to diminished muscle strength, cognitive capacity, and sensory functions, consequently heightening frailty susceptibility (1, 7, 44).

The potential mechanisms underlying the relationship between the TyG index and frailty remain unclear and may involve several mediating factors, including the following: (1) Skeletal muscle serves as the main location for insulin-mediated glucose metabolism (45). Aging, accompanied by diminishing hormone levels and decreased physical activity, induces a decline in skeletal muscle mass and a progressive impairment in peripheral glucose uptake capacity. Consequently, this sequence of events culminates in hyperinsulinemia and the development of IR. Elevated TyG index, utilized as a proxy for IR, may stem from decreased responsiveness to insulin, thereby influencing glucose and fatty acid metabolism, weakening proteolysis in skeletal muscles, disrupting muscle catabolism and quality, and ultimately leading to muscle loss and frailty (2, 12, 24). (2) An increase in the TyG index is correlated with increased levels of chronic inflammation and oxidative stress in the body, causing harm to cells and tissues, impeding the body’s repair and regeneration abilities, thus facilitating the progression of frailty (46–48).

This study reveals a positive linear correlation between the TyG index and frailty, offering a novel perspective for clinical practice. The TyG index is a cost-effective clinical parameter that requires only fasting blood glucose and triglyceride levels from routine tests. It is useful for screening high-risk populations, especially older individuals vulnerable to frailty. Early identification using the TyG index enables healthcare providers to initiate timely interventions and health managements to delay or prevent frailty onset. For patients with elevated TyG index values, personalized intervention plans can be devised, including lifestyle modifications (dietary adjustments and increased physical activity), along with targeted health education and lifestyle counseling to enhance metabolic health and reduce frailty risk. Monitoring changes in the TyG index during follow-up assessments facilitates the evaluation of intervention effectiveness and enables prompt adjustments to treatment and care plans.

## 6 Strengths and limitations

In this pioneering study on middle-aged and older individuals in China, we examined the link between the TyG index and frailty for the first time. Our cross-sectional analysis found a significant positive correlation between higher TyG index levels and frailty occurrence, which remained robust after adjusting for age, gender, lifestyle variables, and biochemical markers.

However, this study is subject to limitations. Firstly, this cross-sectional study does not enable the establishment of a causal relationship between the TyG index and frailty. Secondly, the evaluation of frailty is based on survey data, which can be susceptible to information bias. Lastly, while attempts are made to address confounding factors, it is difficult to entirely exclude their influence on the study findings.

## 7 Conclusion

To summarize, a direct linear association is evident between the TyG index and the vulnerability to frailty in the older. An increased risk of frailty is evident in older adults with higher TyG index levels. It is crucial to promptly counsel individuals in this group to adopt lifestyle modifications to prevent and postpone the onset or advancement of frailty.

## Data Availability

The datasets presented in this study can be found in online repositories. The names of the repository/repositories and accession number(s) can be found in the article/Supplementary material.

## Footnotes

^1^ http://charls.pku.edu.cn
