# Supplementary material for: Investigation of the association between the triglyceride-glucose index and the incidence of frailty among middle-aged and older adults: evidence from the China health and retirement longitudinal study
Source: Front Public Health. 2025 Apr 30;13:1548222. doi: 10.3389/fpubh.2025.1548222 (PMC12074911; doi:10.3389/fpubh.2025.1548222)
Supplement: Supplementary file 1 [file Data_Sheet_1.docx]

Supplementary Material

# Supplementary Figures and Tables

## Supplementary table 1 List of 36 variables included in the frailty index

| No | Items description | assignment |
| --- | --- | --- |
|  | CHARLS |  |
| 1 | Self-reported doctor-diagnosed hypertension. | Yes = 1, No = 0 |
| 2 | Self-reported doctor-diagnosed hyperlipidemia | Yes = 1, No = 0 |
| 3 | Self-reported doctor-diagnosed diabetes. | Yes = 1, No = 0 |
| 4 | Self-reported doctor-diagnosed heart disease. | Yes = 1, No = 0 |
| 5 | Self-reported doctor-diagnosed stroke. | Yes = 1, No = 0 |
| 6 | Self-reported doctor-diagnosed cancer. | Yes = 1, No = 0 |
| 7 | Self-reported doctor-diagnosed arthritis. | Yes = 1, No = 0 |
| 8 | Self-reported doctor-diagnosed chronic lung disease. | Yes = 1, No = 0 |
| 9 | Self-reported doctor-diagnosed asthma. | Yes = 1, No = 0 |
| 10 | Self-reported emotional and mental problems. | Yes = 1, No = 0 |
| 11 | Self-reported memory-related diseases. | Yes = 1, No = 0 |
| 12 | Self-reported kidney disease. | Yes = 1, No = 0 |
| 13 | Self-reported stomach disease. | Yes = 1, No = 0 |
| 14 | Self-reported liver disease. | Yes = 1, No = 0 |
| 15 | Self-reported vision problems. | Yes = 1, No = 0 |
| 16 | Self-reported hearing problems. | Yes = 1, No = 0 |
| 17 | Self-reported general health status. | Poor or fair = 1, excellent, very good, or good = 0 |
| 18 | Difficulty in dressing? | No = 0，Have difficulties can be completed=0.25  Have difficulties and need help=0.75,Yes = 1 |
| 19 | Difficulty in bathing or showering? | No = 0，Have difficulties can be completed=0.25  Have difficulties and need help=0.75,Yes = 1 |
| 20 | Difficulty in eating? | No = 0，Have difficulties can be completed=0.25  Have difficulties and need help=0.75,Yes = 1 |
| 21 | Difficulty in getting in and out of bed? | No = 0，Have difficulties can be completed=0.25  Have difficulties and need help=0.75,Yes = 1 |
| 22 | Difficulty in using the toilet? | No = 0，Have difficulties can be completed=0.25  Have difficulties and need help=0.75,Yes = 1 |
| 23 | Difficulty in managing finances? | No = 0，Have difficulties can be completed=0.25  Have difficulties and need help=0.75,Yes = 1 |
| 24 | Difficulty in taking medicine? | No = 0，Have difficulties can be completed=0.25  Have difficulties and need help=0.75,Yes = 1 |
| 25 | Difficulty in shopping? | No = 0，Have difficulties can be completed=0.25  Have difficulties and need help=0.75,Yes = 1 |
| 26 | Difficulty in cooking? | No = 0，Have difficulties can be completed=0.25  Have difficulties and need help=0.75,Yes = 1 |
| 27 | Difficulty in doing housework? | No = 0，Have difficulties can be completed=0.25  Have difficulties and need help=0.75,Yes = 1 |
| 28 | Difficulty in walking 100 meters? | No = 0，Have difficulties can be completed=0.25  Have difficulties and need help=0.75,Yes = 1 |
| 29 | Is it difficult for you to stand up after sitting on a chair for a long time? | No = 0，Have difficulties can be completed=0.25  Have difficulties and need help=0.75,Yes = 1 |
| 30 | Is it difficult for you to climb several floors continuously? | No = 0，Have difficulties can be completed=0.25  Have difficulties and need help=0.75,Yes = 1 |
| 31 | Is it difficult for you to lift something weighing 10 catties? | No = 0，Have difficulties can be completed=0.25  Have difficulties and need help=0.75,Yes = 1 |
| 32 | Is it difficult for you to pick up a small coin from the table? | No = 0，Have difficulties can be completed=0.25  Have difficulties and need help=0.75,Yes = 1 |
| 33 | Is it difficult for you to bend over, bend your knees or squat? | No = 0，Have difficulties can be completed=0.25  Have difficulties and need help=0.75,Yes = 1 |
| 34 | Is it difficult for you to stretch your arm upward along your shoulder? | No = 0，Have difficulties can be completed=0.25  Have difficulties and need help=0.75,Yes = 1 |
| 35 | Depression score. | CESD-10≥10 =1, <10 =0 |
| 36 | Cognition: (memory test score + orientation test score) / 15 | Continuous, ranging from 0 to 1 |

Note: The memory score is the average number of words not recalled in the immediate and delayed word recall tasks. The memory score ranges from 0 to 10. The orientation test score includes four questions about the day of the week, month, date, year, and season. One point is given for each incorrect answer, ranging from 0 to 5.

## Supplementary Figures


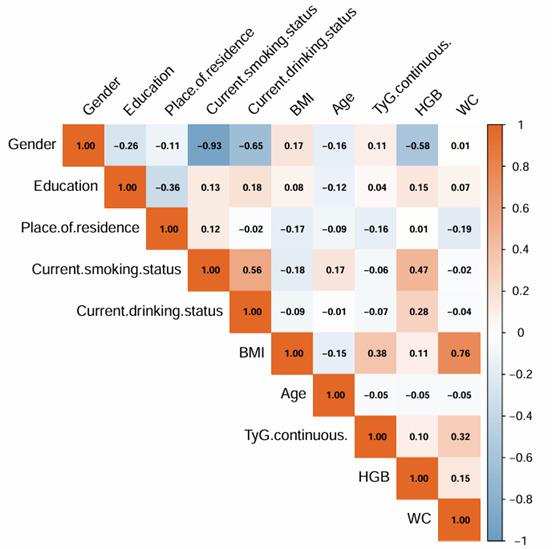


## Supplementary Figure 1 Visualization of the correlation matrix (TyG as a continuous variable)

**
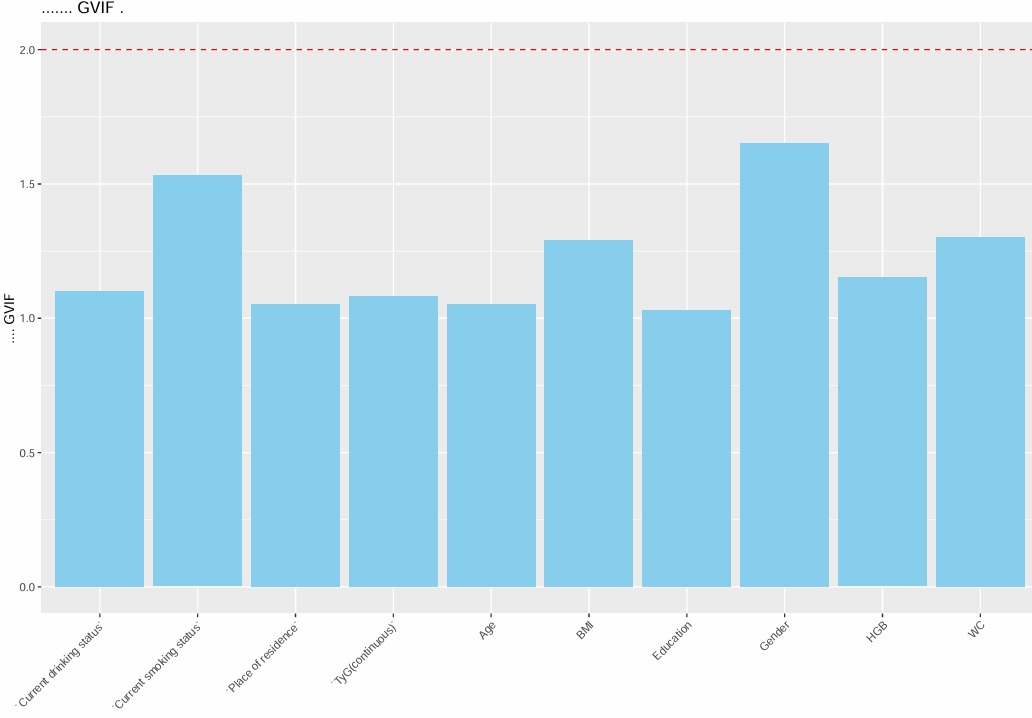
**

**Supplementary Figure 2 Visualization of multicollinearity (TyG as a continuous variable).**

**
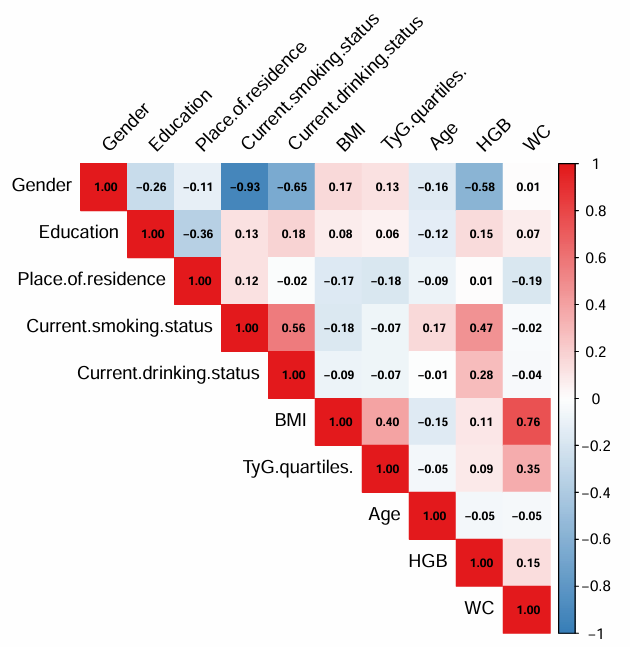
**

**Supplementary Figure 3 Visualization of the correlation matrix (TyG as a quartiles variable)**


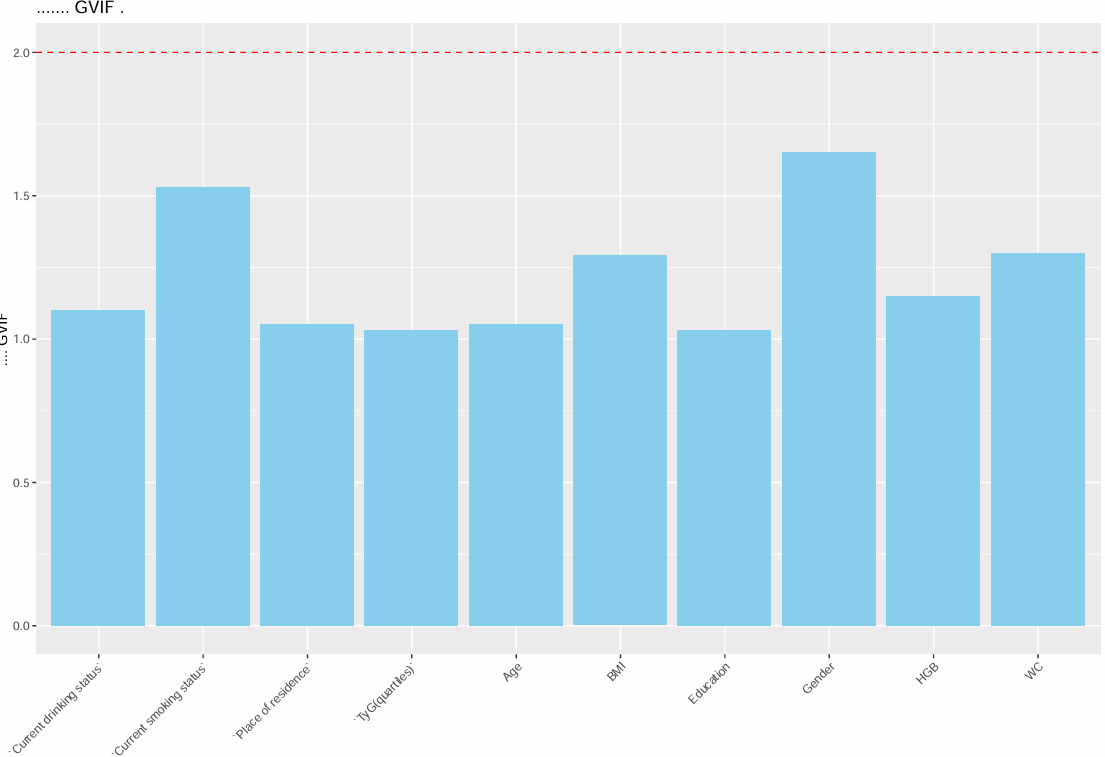


**Supplementary Figure 4 Visualization of multicollinearity (TyG as a quartiles variable).**
